# A nursing and midwifery training program in Kathmandu on antimicrobial resistance and stewardship and infection prevention and control: a qualitative and quantitative outcomes and process evaluation

**DOI:** 10.3389/fpubh.2025.1497335

**Published:** 2025-01-23

**Authors:** Jamuna Tamrakar Sayami, Rakchya Amatya, Kshitij Karki, Deepak Bajracharya, Basudha Shrestha, Sreenidhi Srinivasan, Tyler Prentiss, Anita Shallal, Marcus Zervos, Katie Latack, Linda Kaljee

**Affiliations:** ^1^National Centre for Health Profession Education, Kathmandu, Nepal; ^2^Group for Technical Assistance, Lalitpur, Nepal; ^3^Kathmandu Model Hospital, Kathmandu, Nepal; ^4^Henry Ford Health, Global Health Initiative, Detroit, MI, United States; ^5^Henry Ford Health, Division of Infectious Diseases, Detroit, MI, United States; ^6^Henry Ford Health, Public Health Sciences, Detroit, MI, United States

**Keywords:** antimicrobial stewardship, infection prevention and control, program evaluation, South Asia, Nepal

## Abstract

**Background:**

Low- and middle-income countries (LMICs) are disproportionately affected by antimicrobial resistance (AMR). Nurses and midwives are essential to a holistic approach to AMR stewardship (AMS) and IPC within LMICs.

**Objective:**

(1) Adapt AMS and IPC training programs and practice guidelines for community- and hospital-based nurses and midwives in Nepal; (2) pilot and conduct training outcome and process evaluations.

**Design:**

A one-day training was developed through partnerships between Henry Ford Health and nursing and midwifery organizations and teaching facilities in Nepal. Quantitative outcome and process evaluations were conducted. Qualitative process evaluation interviews were conducted with purposefully selected trainees.

**Setting(s):**

Trainees worked in healthcare facilities in Kathmandu Valley.

**Participants:**

A total of 126 nurses and midwives participated in the training and the quantitative evaluation. Eighteen trainees participated in the process evaluation interviews.

**Methods:**

The 10-module program was adapted from AMS and IPC materials from the World Health Organization and the Nepal Ministry of Health and Population, and curricula from previous AMS studies in Nepal. Key outcomes were AMS and IPC knowledge, and decision-making about empirical dispensing of antibiotics. The process evaluation focused on training content, integration into practice, implementation barriers, and recommendations for dissemination. Quantitative data analysis included descriptive and bivariate analysis. Qualitative data analysis included coding, searches, review of coded texts, and identification of patterns and themes.

**Results:**

AMS and AMR knowledge increased at immediate [1.40 (1.06–1.74) CI 95%] and six-month post-training [0.71 (0.35–1.08) CI 95%]. IPC knowledge also increased at immediate [0.79 (0.55–1.03) CI 95%] and six-month post-training [0.72 (0.49–0.96) CI 95%]. At immediate post-training, an increasing number of respondents indicated that they would not dispense antibiotics for adults [14.74% (4.88, 24.60%) CI 95%] and children [8.13% (−1.88, 18.14%) CI 95%] with fever and sore throats, and for non-pregnant women with burning sensation when urinating [10.69% (0.68%, 20.71%) CI 95%]. Process evaluation data indicated positive responses to the training content and relevancy to practice.

**Conclusion:**

The AMS-IPC training increased knowledge and decreased intentions for dispensing antibiotics. Participants provided concrete examples of implementation of learnings into practice. Trainings will be adapted to address identified content needs and challenges to implementation.

## 1 Introduction

Antibiotic use has seen an exponential increase in low- and middle-income countries (LMICs) over the last decade and low-resource settings are disproportionately affected by antimicrobial resistance (AMR) (1). The increase in antibiotic use has been enabled by rising incomes, availability of cheaper generic antibiotics, as well as policy-related barriers that include limited national guidelines, unregulated over-the-counter pharmacy dispensing practices, and inappropriate antibiotic use in health care (2). In addition, international travel and migration contribute to the global spread of resistant pathogens (3). These issues are further heightened due to limited resources in laboratories, healthcare facilities, and communities to establish and sustain stewardship and infection control and prevention (IPC) programs. Furthermore, data indicate that AMR burden has increased during the COVID-19 pandemic due to long-term hospitalization, resource reallocation to address the pandemic, challenges to use of appropriate IPC strategies with increased personal protection equipment (PPE) burden, and lack of information and misuse of antibiotics to treat febrile and pulmonary symptoms (4–8).

During surveillance at a 125-bed hospital in Kathmandu in 2016–2017, 71 patients were hospitalized with multidrug resistant (MDR) infections. MDR bacteria included extended spectrum beta lactamase (ESBL), carbapenem (CRE), or colistin resistance. A majority of these infections were determined to be community-acquired (9). Ansari et al., found that among community-associated isolates in Nepali hospitalized patients, 78% of the total isolates were MDR. ESBL, MBL and AmpC production was found in 24, 15, and 9% of isolates, respectively (10). In another study focused on *Acinetobacter* spp., MDR was identified in 30.2% (110) and extensively drug resistance (XDR) in 23.9% (87) of samples. Overall, approximately one in five patients died (11).

There is a broad array of antimicrobial stewardship (AMS) programs available. However, AMS programs need to be developed and/or adapted to fit within existing health delivery and education networks and meet locally defined needs related to antibiotic use and resistance patterns (12). A key element of translation of policy to programs and program sustainability is early and ongoing engagement of policy leaders, health administrators, healthcare providers, and local communities. AMS programs are often focused on physicians as prescribers of antibiotics in LMIC. In reviews of stewardship programs, few were specifically tailored to nurses, midwives, pharmacists, and students (13, 14). The American Nursing Association has identified nurses as educators and advocates in hospitals, clinics, and communities to support AMS programs (15). Nurses and midwives contribute significantly to the appropriate delivery of antibiotics to maximize their effectiveness, monitoring of patient progress and potential reactions to prescribed medications, and patient education (16).

Infection prevention and control (IPC) has been identified as a key intervention when paired with stewardship programs to reduce the long-term effects of AMR (17). Nurses play an important role in overseeing IPC strategies to reduce the spread of resistant pathogens. Healthcare-associated infections (HAI) have an estimated prevalence in LMIC of anywhere from 2 to 20 times greater than in high-income settings (18). Within health facilities, AMR pathogens are spread by healthcare personnel, patients, and their visitors. Inadequate IPC strategies compromise the quality of healthcare service delivery and increase the incidence of HAI (19). AMS and IPC require a holistic and multidisciplinary approach to ensure effective program implementation and execution needed to combat the spread of antibiotic-resistant pathogens (20).

The objectives of the current project included: (1) Adaptation of existing AMS and IPC training programs and practice guidelines to meet the needs and challenges for implementing AMS and IPC programs among nurses and midwives in Nepal; (2) Piloting the AMS-IPC training with community- and hospital-based nurses and midwives in Kathmandu Valley; and (3) Conducting a longitudinal outcome evaluation and mixed methods process evaluation.

## 2 Materials and methods

### 2.1 Development and implementation of the AMS-IPC training program for nurses and midwives

The development of the AMS-IPC training program was a collaboration between Henry Ford Health Global Health Initiative and Division of Infectious Disease, the Group for Technical Assistance (Kathmandu-based NGO), the National Centre for Health Profession Education, the Nepal Nursing IPC Association, and the Midwifery Society of Nepal. Content was obtained from AMS and IPC materials and toolkits from the World Health Organization and the Nepal Ministry of Health and Population, and curricula and data from previous AMR and AMS studies in Nepal (9, 21–24).

The final program was organized on a power point presentation and included case studies as a group activity. The program included 10 modules: (1) Introduction to Stewardship and Nursing and Midwifery Practice; (2) Antimicrobial Resistance and Stewardship; (3) Antimicrobial Resistance in Nepal; (4) The Role of Nurses and Midwives in Community- and Hospital-based Stewardship; (5) Antibiotic and OTC Guidelines for Community-based Nurses and Midwives; (6) Case Studies on Use of OTC Guidelines; (7) Infection Prevention and Control; (8) Healthcare Waste Management; (9) AMS and IPC Community Education and Outreach; and (10) Course Summary and Overview.

The program was implemented in September 2021 in five groups due to COVID-19 restrictions on numbers of people at a gathering. A total of 126 nurses and midwives were trained. Most of the modules were presented by Nepal experts in nursing, midwifery, IPC, and microbiology with remaining modules conducted by the U.S. principal investigator.

Participants for the training were purposefully selected to ensure representation of disciplines (nursing and midwifery), workplaces and positions, education, and specialists (see Demographics). All participants worked within Kathmandu Valley as that area was the focus of the research. Nurses and midwives were similarly selected from the trainees to participate in the qualitative process evaluation interviews.

### 2.2 Outcome evaluation survey

#### 2.2.1 Data collection and management

The evaluation survey was developed from previous AMR and IPC surveys conducted in Nepal and information included in the training program. Data were collected by pen and paper prior to the training (baseline) and then the same day for the immediate post-training. The survey was sent out to participants through email for collection of the six-month post-training data. Survey data were entered into REDCap, a secure web application for building and managing online surveys and databases (25). The outcome evaluation survey data presented here included sections on respondent demographics, education, and employment, AMS and IPC knowledge and perceptions of appropriate empiric antibiotic dispensing for common symptoms among children and adults.

#### 2.2.2 Outcome evaluation sample size

The sample size is based on the primary hypothesis that participants will have increased knowledge about AMS, AMR, and IPC at post-training compared to baseline. For conduct of a t-test (continuous variable) with an effect size of 0.4, power 0.9 and *α* = 0.05, a minimum sample size of 110 is needed. Total trainees completing baseline and immediate- and six-month post-training was 126, which is sufficient for the proposed analysis and to assess a medium effect size.

#### 2.2.3 Outcome evaluation data analysis

Outcome evaluation data was reviewed and cleaned by the study team. Variables were created for knowledge scales and any missing data and outliers were removed. Analysis focused on descriptive statistics and bivariate analysis. The mean difference and 95% confidence intervals were calculated to assess differences between time points within participant place of employment. Differences in proportions and 95% confidence intervals were calculated to assess the differences in time points within dispensing decisions. Analyses were performed using SAS 9.4 (SAS Institute Inc., Cary, NC, United States) and SPSS 25 (IBM Corp, Armonk, NY, United States).

### 2.3 Process evaluation

#### 2.3.1 Data collection and management

The process evaluation data included 10 quantitative items on the immediate- and six-month post-training evaluation survey. These items assessed trainees’ perceptions of the need for AMS and IPC in their clinical practice, the practicality of introducing changes, and their confidence in introducing AMS and IPC at their workplace.

To obtain more in-depth understanding of trainees’ perceptions of the program and how they implemented learnings in their workplace, qualitative data were collected from a purposeful sample of nurse and midwife participants. Selection was designed to include both hospital- and community-based health workers. A total of 18 trainees were interviewed. An interview guide included items on training content and delivery, training dissemination to peers, integration of information learned into practice, barriers to implementation of AMS and IPC programs in the workplace, and recommendations for future dissemination of the training in Nepal.

Qualitative interviews were conducted both virtually and in-person and in English and/or Nepali depending on the preference of the respondent. All interviews were audio recorded, transcribed, and as necessary translated from Nepali to English. Transcribed data were uploaded into a qualitative data management program (Dedoose). A coding dictionary was developed based on the interview guide topics and emergent themes. Coding was conducted by the project principal investigator.

#### 2.3.2 Process evaluation data analysis

The quantitative process evaluation data analysis included descriptive statistics (response numbers and percentages) at immediate and six-month post-training data points. The qualitative coded data were reviewed to identify cogent themes and patterns within coded text. The themes were documented in a table format with illustrative texts from the transcripts. For the current paper, both the quantitative and qualitative data are presented under subheadings related to training content and delivery, perceived need for AMS and IPC education, implementation of knowledge into practice, implementation barriers, and perspectives on future program dissemination.

### 2.4 Ethical approval

The study was approved by the Nepal Human Research Council (NHRC) [reference number 2039] and the Henry Ford Health Institutional Review Board [reference number 14112]. All participants completed a written consent form prior to data collection.

## 3 Results

### 3.1 Outcome evaluation

#### 3.1.1 Demographics

A total of 126 nurses were trained. Gender data was collected for 124 participants, with 100% (*n* = 124) reporting as female. The majority of participants had a bachelor’s degree in nursing (*n* = 83, 69.7%), with participants also having Auxiliary Nurse Midwifery diplomas (*n* = 5, 4.2%), certificate-level training in nursing (*n* = 19, 16%), and master’s degree training (*n* = 9, 7.6%). Participants worked in a variety of health settings in Nepal, including government hospitals, private hospitals, primary health care centers, and universities and colleges. In terms of positions, 51 (43.2%) reported being a staff nurse, 23 (19.5%) participants were nursing supervisors/officers, and 18 (15.3%) participants were ward charge nurses. The trainings also included 7 (5.9%) auxiliary nurse midwives and 3 (2.5%) midwife officers. Primary specialties of participants included internal medicine, general surgery, obstetrics & gynecology, and infectious disease (Table 1).

**Table 1:** Demographics of participants in AMR-IPC program outcome evaluation study (baseline)

Item	Response options	
Gender (*N* = 124)	Female	100% (124)
Age (mean) (*N* = 119)		36.1 (SD 9.9) Range 20 to 66
Education (*N* = 119)	ANM	4.2% (5)
	Certificate level nursing	16.0% (19)
	Bachelor nursing midwife	69.7% (83)
	MA nursing midwife	7.6% (9)
	other	2.5% (3)
Current employment (*N* = 124)	Government hospital	24.2% (30)
	Private hospital	21.8% (27)
	Non-profit hospital	25.8% (32)
	Public health center/primary health center	12.1% (15)
	University or college	14.5% (18)
	Other	1.6% (2)
Current position (*N* = 118)	ANM or senior ANM	5.9% (7)
	Staff nurse	43.2% (51)
	Nursing supervisor/officer	19.5% (23)
	Matron	2.5% (3)
	Midwife officer	2.5% (3)
	Ward in charge/in charge	15.3% (18)
	Nurse coordinator	2.5% (3)
	Nursing instructor	5.1% (6)
	Other	3.4% (4)

#### 3.1.2 AMR/AMS and IPC knowledge

**Figure 1:**
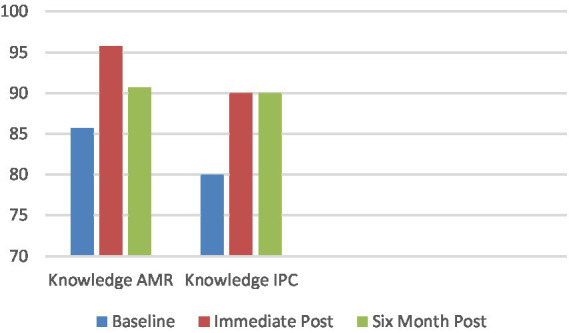
Knowledge scores at baseline, immediate post and 6 months post (percentage correct) (*N* = 126).

AMS and AMR knowledge increased at immediate [1.40 (1.06–1.74) CI 95%] and six-month post-training [0.71 (0.35–1.08) CI 95%]. IPC knowledge also increased at immediate [0.79 (0.55–1.03) CI 95%] and six-month post-training [0.72 (0.49–0.96) CI 95%] (Figure 1). In relation to AMR/AMS knowledge, there were significant differences between baseline and immediate post scores for participants employed at non-profit, public, and government hospitals, as well as at public health centers and universities/colleges. Additionally, there was a significant difference between non-profit hospital and baseline and 6-month scores. In relation to IPC knowledge, there were significant differences between baseline and immediate post scores for participants employed at non-profit hospitals, public health centers, and universities/colleges. There were significant differences in scores between baseline and 6-month post intervention for government hospital, non-profit hospital, and public health center employees (Table 2).

**Table 2:** Knowledge of antimicrobial resistance and stewardship (AMR/AMS) and infection prevention and control (IPC) at baseline, immediate post, and 6-month post intervention by place of employment.

Knowledge AMR					
Current employment		Base	Intermediate	6-months	Mean difference (95% CI)
Government hospital		(*N* = 30)	(*N* = 30)	(*N* = 30)	0.87 (0.37–1.36)^A^ 0.50 (−0.11–1.11)^B^
	N	30	30	30	
	Mean (SD)	12.7 (1.34)	13.6 (0.73)^2^	13.2 (1.03)	
Private hospital		(*N* = 27)	(*N* = 27)	(*N* = 27)	1.00 (0.41–1.59)^A^ 0.56 (−0.24–1.35)^B^
	N	27	27	27	
	Mean (SD)	12.1 (1.77)	13.1 (1.35)^2^	12.7 (2.07)	
Non-profit hospital		(*N* = 32)	(*N* = 32)	(*N* = 32)	1.56 (0.85–2.28)^A^ 0.75 (0.12–1.38)^B^
	N	32	32	32	
	Mean (SD)	11.7 (1.82)	13.3 (0.92)^3^	12.4 (1.16)^1^	
Public health center/primary health center		(*N* = 15)	(*N* = 15)	(*N* = 15)	2.20 (0.92–3.48)^A^ 0.73 (−0.65–2.11)^B^
	N	15	15	15	
	Mean (SD)	11.5 (2.00)	13.7 (0.72)^2^	12.2 (1.97)	
University or college		(*N* = 18)	(*N* = 18)	(*N* = 18)	1.50 (0.29–2.71)^A^ 0.89 (−0.51–2.28)^B^
	N	18	18	18	
	Mean (SD)	11.8 (2.04)	13.3 (1.03)^1^	12.7 (1.23)	
Knowledge IPC					
Government hospital		(*N* = 30)	(*N* = 30)	(*N* = 30)	0.33 (−0.05–0.72)^A^ 0.60 (1.2–1.08)^B^
	N	30	30	30	
	Mean (SD)	6.9 (1.08)	7.3 (0.87)^2^	7.5 (0.68)	
Private hospital		(*N* = 27)	(*N* = 27)	(*N* = 27)	0.33 (−0.23–0.89)^A^ 0.29 (−0.14–0.73)^B^
	N	27	27	27	
	Mean (SD)	7.0 (1.34)	7.4 (0.97)^2^	7.3 (0.83)	
Non-profit hospital		(*N* = 32)	(*N* = 32)	(*N* = 32)	1.19 (0.67–1.7)^A^ 1.13 (0.62–1.63)^B^
	N	32	32	32	
	Mean (SD)	5.9 (1.29)	7.1 (0.75)^3^	7.1 (0.88)^1^	
Public health center/primary health center		(*N* = 15)	(*N* = 15)	(*N* = 15)	1.27 (0.53–2.01) 0.93 (0.01–1.86)^B^
	N	15	15	15	
	Mean (SD)	6.2 (1.47)	7.5 (0.64)^2^	7.1 (1.19)	
University or college		(*N* = 18)	(*N* = 18)	(*N* = 18)	1.00 (0.29–1.70)^A^ 0.61 (−0.05–1.27)^B^
	N	18	18	18	
	Mean (SD)	5.9 (0.80)	6.9 (1.00)^1^	6.6 (1.25)	

A Comparison of base and intermediate.

B Comparison of base and 6 month.

1 *p < 0.05.*

2 *p < 0.01.*

3 *p < 0.001.*

#### 3.1.3 Perceptions of empiric antibiotic dispensing practices for adults and children

Participants responded to a series of scenarios of a patient or the parent of a patient with symptoms that are frequently associated with antibiotic dispensing, prescribing, and use in Nepal. These questions were previously used in a study of dispensing practices of community pharmacists in Nepal (26). Symptoms included either a fever and sore throat or diarrhea, as well as pregnant and non-pregnant women presenting with burning sensations while urinating. At immediate post-training, an increasing number of respondents indicated that they would not dispense antibiotics for adults [14.74% (4.88, 24.60%) CI 95%] and children [8.13% (−1.88, 18.14%) CI 95%] with fever and sore throats, and for non-pregnant women with burning sensation when urinating [10.69% (0.68%, 20.71%) CI 95%]. There was a significant decrease in number of respondents reporting they would not dispense antibiotics for child diarrhea between baseline and six-months post-training [−9.41% (−19.54, 0.71%) CI 95%] (Table 3).

**Table 3:** Decisions regarding antibiotic use for adults and children presenting with common symptoms [cough, fever, diarrhea, burning sensation during urination] (*N* = 126).

	Base	Intermediate	6-months	Difference (95% CI) Base and intermediate	Difference (95% CI) Base and 6 month
Adult fever and sore throat n (%)					
Would not dispense	16 (12.9%)	34 (27.6%)^3^	32 (25.8%)	14.74% (4.88, 24.6%)	12.9% (3.2, 22.61%)
Not likely	21 (16.9%)	15 (12.2%)	12 (9.7%)	−4.74% (−13.52, 4.04%)	−7.26% (−15.66, 1.15%)
Likely	31 (25.0%)	13 (10.6%)	20 (16.1%)	−14.43% (−23.79, −5.07%)	−8.87% (−18.87, 1.13%)
Very likely	16 (12.9%)	5 (4.1%)	6 (4.8%)	−8.84% (−15.69, −1.98%)	−8.06% (−15.07, −1.06%)
Not applicable	40 (32.3%)	56 (45.5%)	54 (43.5%)	13.27% (1.22, 25.32%)	11.29% (−0.7, 23.28%)
Missing	2	3	2		
Child fever and sore throat n (%)					
Would not dispense	20 (16.3%)	30 (24.4%)^2^	25 (20.5%)	8.13% (−1.88, 18.14%)	4.23% (−5.45, 13.92%)
Not likely	32 (26.0%)	24 (19.5%)	16 (13.1%)	−6.5% (−16.95, 3.94%)	−12.9% (−22.7, −3.1%)
Likely	23 (18.7%)	12 (9.8%)	22 (18.0%)	−8.94% (−17.6, −0.28%)	−0.67% (−10.36, 9.03%)
Very likely	10 (8.1%)	2 (1.6%)	6 (4.9%)	−6.5% (−11.83, −1.18%)	−3.21% (−9.38, 2.96%)
Not applicable	38 (30.9%)	55 (44.7%)	53 (43.4%)	13.82% (1.83, 25.82%)	12.55% (0.55, 24.55%)
Missing	3	3	4		
Adult diarrhea n (%)					
Would not dispense	28 (22.8%)	27 (22.0%)	19 (15.8%)	−0.81% (−11.23, 9.6%)	−6.93% (−16.81, 2.95%)
Not likely	26 (21.1%)	23 (18.7%)	18 (15.0%)	−2.44% (−12.42, 7.54%)	−6.14% (−15.78, 3.5%)
Likely	20 (16.3%)	11 (8.9%)	14 (11.7%)	−7.32% (−15.56, 0.93%)	−4.59% (−13.28, 4.1%)
Very likely	8 (6.5%)	3 (2.4%)	8 (6.7%)	−4.07% (−9.21, 1.08%)	0.16% (−6.08, 6.4%)
Not applicable	41 (33.3%)	59 (48.0%)	61 (50.8%)	14.63% (2.5, 26.77%)	17.5% (5.28, 29.72%)
Missing	3	3	6		
Child diarrhea n (%)					
Would not dispense	32 (25.8%)	29 (23.4%)	20 (16.4%)^1^	−2.42% (−13.14, 8.3%)	−9.41% (−19.54, 0.71%)
Not likely	29 (23.4%)	21 (16.9%)	16 (13.1%)	−6.45% (−16.41, 3.5%)	−10.27% (−19.83, −0.71%)
Likely	19 (15.3%)	9 (7.3%)	16 (13.1%)	−8.06% (−15.88, −0.25%)	−2.21% (−10.93, 6.51%)
Very likely	4 (3.2%)	3 (2.4%)	12 (9.8%)	−0.81% (−4.93, 3.31%)	6.61% (0.48, 12.74%)
Not applicable	40 (32.3%)	62 (50.0%)	58 (47.5%)	17.74% (5.69, 29.79%)	15.28% (3.19, 27.38%)
Missing	2	2	4		
Woman with burning sensation when urinating n (%)					
Would not dispense	19 (15.3%)	32 (26.0%)^2^	16 (13.0%)	10.69% (0.68, 20.71%)	−2.31% (−11.01, 6.38%)
Not likely	18 (14.5%)	21 (17.1%)	22 (17.9%)	2.56% (−6.53, 11.65%)	3.37% (−5.81, 12.55%)
Likely	37 (29.8%)	16 (13.0%)	32 (26.0%)	−16.83% (−26.84, −6.82%)	−3.82% (−15, 7.36%)
Very likely	15 (12.1%)	5 (4.1%)	10 (8.1%)	−8.03% (−14.75, −1.31%)	−3.97% (−11.47, 3.53%)
Not applicable	35 (28.2%)	49 (39.8%)	43 (35.0%)	11.61% (−0.12, 23.34%)	6.73% (−4.83, 18.3%)
Missing	2	3	3		
Pregnant woman with burning sensation when urinating n (%)					
Would not dispense	44 (35.5%)	34 (27.6%)	26 (21.0%)	−7.84% (−19.39, 3.71%)	−14.52% (−25.57, −3.46%)
Not likely	24 (19.4%)	18 (14.6%)	26 (21.0%)	−4.72% (−14.07, 4.63%)	1.61% (−8.37, 11.6%)
Likely	13 (10.5%)	12 (9.8%)	11 (8.9%)	−0.73% (−8.25, 6.79%)	−1.61% (−8.97, 5.74%)
Very likely	5 (4.0%)	3 (2.4%)	4 (3.2%)	−1.59% (−6, 2.81%)	−0.81% (−5.46, 3.85%)
Not applicable	38 (30.6%)	56 (45.5%)	57 (46.0%)	14.88% (2.91, 26.85%)	15.32% (3.37, 27.27%)
Missing	2	3	2		

1 *p < 0.05.*

2 *p < 0.01.*

3 *p < 0.001.*

### 3.2 Process evaluation

#### 3.2.1 Training content and delivery

A majority of respondents at both immediate and six-month post-training agreed or strongly agreed that the content fit their day-to-day clinical needs (100% [126]/100% [126]), was practical and adaptable (96.9% [122]/94.4% [118]) and fit within other programs and policies at their workplaces (100% [126]/100% [124]) (Table 4).

**Table 4:** Quantitative survey process evaluation items at post-immediate and 6-months post (*N* = 126).

	Immediate-post	6-month post
I feel that the intervention content is tailored to my needs in my day-to-day work as a health practitioner, n (%)		
Agree	27 (21.4%)	58 (46.0%)
Strongly agree	99 (78.6%)	68 (54.0%)
The training content is practical and can be easily adapted to my work setting, n (%)		
Disagree	4 (3.2%)	7 (5.6%)
Agree	55 (43.7%)	70 (56.0%)
Strongly agree	67 (53.2%)	48 (38.4%)
Missing	0	1
I feel that there is a need for antimicrobial resistance and stewardship training for providers in my position, n (%)		
Strongly disagree	0	1 (0.8%)
Agree	27 (21.4%)	26 (20.6%)
Strongly agree	99 (78.6%)	99 (78.6%)
I feel that there is a need for infection prevention and control training for providers in my position, n (%)		
Agree	29 (23.2%)	16 (12.7%)
Strongly agree	96 (76.8%)	110 (87.3%)
Missing	1	0
I feel that I can contribute to future AMR, AMS, IPC training programs, n (%)		
Disagree	0	2 (1.6%)
Agree	42 (33.3%)	64 (50.8%)
Strongly agree	84 (66.7%)	60 (47.6%)
I feel that the training I have will make a long-term change in the practices at my place of work, n (%)		
Disagree	0	3 (2.4%)
Agree	55 (43.7%)	66 (52.8%)
Strongly agree	71 (56.3%)	56 (44.8%)
Missing	0	1
I feel that I can effectively implement the skills and knowledge I learned in the AMR, AMS, IPC training in my place of work, n (%)		
Disagree	0	1 (0.8%)
Agree	47 (37.6%)	68 (54.0%)
Strongly agree	78 (62.4%)	57 (45.2%)
Missing	1	0
I feel that the AMR, AMS, and IPC training is something that is needed at this time, n (%)		
Strongly disagree	0	1 (0.8%)
Disagree	1 (0.8%)	1 (0.8%)
Agree	23 (18.3%)	49 (39.5%)
Strongly agree	102 (81.0%)	73 (58.9%)
Missing	0	2
I feel that what I learned in the AMR, AMS, and IPC training fits into other programs and policies at my place of work, n (%)		
Disagree	0	4 (3.2%)
Agree	54 (42.9%)	74 (59.7%)
Strongly agree	72 (57.1%)	46 (37.1%)
Missing	0	2
I have had the opportunity to give feedback to the trainers about the program, n (%)		
Disagree	4 (3.2%)	11 (8.9%)
Agree	68 (54.0%)	77 (62.1%)
Strongly agree	54 (42.9%)	36 (29.0%)
Missing	0	2

Case studies were considered one of the most valuable parts of the training. Participants stated that the case studies provided opportunity for critical thinking and relating training information to their practice.

*…as I mentioned earlier, I felt it would be more effective if you could add more case studies. We could then have more discussion on different cases and the ways to deal with them.* (nurse, non-profit hospital, 27Oct2021)

One respondent had more specific recommendations regarding case studies. She suggested that it would be useful to discuss potential outcomes from the case studies based on different treatment modalities.

*We could make the case studies more effective by doing it in the comparative way. For example, in case of pneumonia, we can present three scenarios: one who receives home remedies, second for the antibiotic treatment and other for the normal medicine. We can compare their effectiveness as well as side effects.* (midwife, Midwife Society of Nepal [MIDSON] 14Mar2022)

A few respondents wanted more information about different generations of antibiotics.

*There was a section on the different generations of antibiotics. We did not have much knowledge on this topic so it would be better if we could learn in detail about this topic. I think most of the doctors nowadays use third generation antibiotics so in that case if we are aware about the topic then we can discuss it with the doctors.* (hospital-based nurse, Society of Cardiothoracic Vascular Nurses of Nepal [SCTVN] 16Mar2022)

One respondent noted that she appreciated learning information from individuals from different disciplines and types of health facilities. A few other trainees also found useful the information on the roles for nurses and midwives within stewardship programs.

*They [trainers] were from different sectors, and they shared their own experiences, so it was quite fruitful…. In addition, there were almost 25 participants from different sectors, so they shared their own experiences and knowledge. That proved to be more fruitful for us as well*. (midwife, MIDSON 24Oct2021)

*I think the module on the role of nurses and midwives in hospital- and community-based stewardship was useful. We nurses must administer antibiotics to patients at the correct dose and at the appropriate time, as directed by the doctors…* (community nurse, Primary Health Care Centre (PHCC) 14Mar2022)

#### 3.2.2 Perceived need for AMS and IPC education

Most respondents at both immediate and 6-month post training agreed or strongly agreed that there is a need for AMR-AMS (100% [126]/99.2% [125]) and IPC training (100% [126]/100% [126]) among nurses and midwives (Table 4).

In terms of specific information about AMR and stewardship, respondents stated that they learned new information about AMR in Nepal, the importance of timing, dosage, and duration during delivery of antibiotics, and the need for patient education.

*I felt the history and the trend of the AMR was a useful module. We were uninformed of the present scenario of AMR prior to the training, but after receiving the training, we became aware of the significant problem of AMR in Nepal.* (hospital-based nurse, non-profit hospital 27Oct2021)

*We gained extensive information from the training and were able to concentrate on the importance of antibiotic timing, dose, and duration…* (midwife, MIDSON 24Oct2021)

*We were aware of antibiotic resistance, yet we were disoriented while practicing. We failed to tell clients that if they don't take antibiotics on time, resistance will develop…after the training, we could make it a priority again. As a result, it was similar to a refresher training.* (midwife, MIDSON 24Oct2021)

In terms of IPC training modules, most of the respondents stated that they had previously learned most of the content. A few felt that it was a good refresher and that it was important to learn about IPC in the context of stewardship. A couple of respondents wanted more information on correct sample collection, handling, and storage.

*Regarding IPC, we have been frequently reading those topics in our setting especially due to pandemic situation. However, it was useful even though it was repetitive.* (midwife, MIDSON 24Oct2021)

*I think that the (AMS) training should be combined with IPC training. Because IPC is such a broad topic, the training should include the basics of standard precautions, proper sample collection, transportation, handling, and storage. Because everything seems to be linked to samples (sputum, blood, urine), microorganisms, and antibiotics, I believe the training might be integrated accordingly.* (hospital-based nurse, Infection Control Society of Nepal [ICSON] 27Oct2021)

#### 3.2.3 Implementation of AMS and IPC practices

All respondents at both immediate and six-month post training agreed or strongly agreed that they can effectively implement the skills and knowledge from the training in their workplaces (100% [126]/100% [124]). A majority also perceived that they can make long-term changes in practices at work (100% [126]/97.6% [122]) (Table 4).

Respondents provided information about how they implemented information they learned during the training both in their personal lives and in their workplaces. Some trainees noted that they had changed their own practices in terms of use of antibiotics and that they had informed family about risks associated with misuse of antibiotics

*The public themselves are not aware of this issue…So, there should be a public awareness campaign. I myself used antibiotics frequently during my illness. But, after the training I have limited the use of it. I suffered from COVID-19 but I treated myself without using any antibiotics.* (community nurse, PHCC 14Mar2022)

*I used to give antibiotics to my child whenever he got an infection, but after the training, I wait 3-4 days and only give him symptomatic medication. I go to the doctor if the symptom does not improve. I even try to convince my family and neighbors that antibiotics should only be taken when recommended by a doctor.* (hospital-based nurse [SCTVN] 16March2022)

*We are thinking about making a poster/chart about AMR and IPC in our PHCC* (primary health care clinic). (community nurse, PHCC 26Oct2021)

A couple of trainees noted that they were more aware and had changed procedures to ensure patients receive their antibiotics on a timely basis and the correct dosage.

*Some people obtain training but do not put it into practice. Antibiotics are given to patients according to a schedule. If a child receives an antibiotic at 3 p.m., the second dose should be given at 3 a.m. At the time, some of the nurses found it difficult to administer the antibiotic. But, after the training we have strictly provided the antibiotic at the exact time. Also, I am a second line in charge in my ward. Therefore, I am authorized to implement some changes in the ward.* (hospital-based nurse, non-profit hospital 14Mar2022)

*We are more conscious about the doses and time of the antibiotics in case we need to use them.* (midwife, MIDSON 24Oct2021)

Another respondent stated that they had instituted use of generic names for antibiotics to make it easier for all health workers to understand what medicines are being prescribed.

*After the training, we have advocated for use of generic names in critical care. If there is a use of generic names, it would be easy for the nurses and other health professionals to understand what type of medicines are being prescribed and on what basis. So, we have started this practice from critical care and will start it in other departments.* (hospital-based nurse, ICSON 27Oct2021)

#### 3.2.4 Barriers to implementation of AMS programs

Despite positive changes being implemented in the hospital wards and clinics, respondents also discussed numerous barriers to making change. Respondents noted that it is important that those who have authority to make change are educated about AMS. In addition, some respondents discussed resistance to change among management and some physicians.

*I have been working in the hospital for quite a long time. So, whenever I express my opinion or make suggestions to my coworkers, they either agree with me or we have a lively discussion. Newly employed nurses, on the other hand, are not in the same boat. It's possible that their suggestions will go unheeded by the rest of the workforce.* (hospital-based nurse, SCTVN 16March2022)

*Despite the fact that our hospital has an antimicrobial stewardship program, implementing all of the activities is difficult. We realize the urgency of AMR but changing the habits and practices of those in management level and physicians is tough. Antibiotics are frequently prescribed by them since they have been doing so for a long time…* (hospital-based nurse, ICSON 15Mar2022)

Another issue is having the time and the right opportunity to educate patients about use of antibiotics.

*We are often in a rush throughout our shifts and are unable to inform patients about AMR.* (hospital-based nurse, ICSON 27Oct2021)

*I believe that patients may be overloaded with information during the postnatal period, as they will be informed not only about antibiotics, but also about breastfeeding, nutrition, hygiene, and other topics. Another option is to inform them during their antenatal appointment so that we can provide one-on-one counseling …but in practice, this is not the case because there is a high case flow during antenatal visits, making detailed information impossible to convey at that time.* (midwife, MIDSON 24Oct2021)

An often-reported challenge is the ‘culture’ of dispensing and prescribing antibiotics in Nepal. Antibiotics are readily available at clinics and pharmacies, and patients often perceive them as necessary to treat a broad range of symptoms. In addition, respondents noted the need for enforcement of national level policies to decrease access to antibiotics.

*… the patient expresses a need for antibiotics and requests it. They believe that because the antibiotic made them feel better in the past, they will require it again now… we are a little uneasy about this situation.* (community nurse, PHCC 26Oct2021)

*Despite informing my family members or coworkers about the consequences of antibiotic overuse, it is difficult to persuade them to limit their antibiotic use. Most of them believe that their illness/infection can only be cured with antibiotics because they have been taking them for a long time.* (hospital-based nurse, non-profit hospital 27Oct2021)

*Yes, this training would be more successful across the country and around the world because antibiotics are sold by pharmacists in community settings, even for common flu cases. We've noticed that most community members trust pharmacists and simply follow their instructions rather than going to the hospital or having their blood tested and antibiotics prescribed.* (midwife, MIDSON 24Oct2021)

*Antibiotic use should be controlled by government rules or regulations, in my opinion. Government controls on the unreasonable use of antibiotics should be implemented. Even doctors provided antibiotics to the majority of patients during the COVID outbreak without performing a culture test. Although most people are aware that COVID-19 is a viral infection, they use the antibiotic as a treatment.* (hospital-based nurse, non-profit hospital 14Mar2022)

#### 3.2.5 Future program dissemination and expansion

Most respondents at both immediate and six-month post-training agreed or strongly agreed that they felt they can contribute to future AMR, AMS, and IPC training programs (100% [126]/98.4% [124]) (Table 4).

Respondents had a number of suggestions regarding future directions for dissemination of the nursing and midwifery AMS-IPC training program. Suggestions included involvement of national level government, pre-service training, and more specific targeted training for midwives, IPC nurses, and health workers in authority to implement change in their facilities.

*This training is required for health-care providers, and it would be most beneficial if it could be delivered to as many health workers as feasible. If the training could be linked with government-provided training, I believe it might reach a bigger range of health practitioners.* (community nurse, PHCC 26Oct2021)

*I believe that the training or content would be more effective if it were integrated into the nursing school curriculum. As a result, all nurses will be educated on this topic at the university level, potentially reducing the AMR problem to some extent.* (hospital-based nurse, ICSON 27Oct2021)

*We can integrate this program with the Aama Surakshya Program, the government program to improve safe motherhood. That way, we can orient the large group of midwives as well as FCHV (Female Community Health Volunteers).* (midwife, MIDSON 14Mar2022)

*…it would be better to provide training to certain designated nurses rather than providing it to all the nurses. In Nepal, the patient-to-nurse ratio is low, hence the majority of nurses are occupied in their wards. They were all unable to focus and advocate for the hospital's AMR problem…I believe that training should be provided to those specific nurses who have the authority to create positive changes at their level, as well as to train other nurses*. (hospital-based nurse, ICSON 27Oct2021)

*Because IP (infection prevention) nurses are continuously working on the surveillance and intervention regarding IPC such as hand hygiene, health care associated infection etc., it would be better to provide training to those nurses if available in the hospital. In case those IPC nurses are not available, I think we can advocate on the need and importance of IPC nurses in the hospital.* (hospital-based nurse, ICSON 27Oct2021)

Most of the respondents discussed the need for training across all health disciplines, as well as the need for community education about AMS and IPC. Some suggested a single training for all health workers within a single health facility. Others mentioned specific groups, such as community pharmacists, which would benefit from AMS and IPC programs.

*Regarding the challenges, I believe that bringing together trainees from many fields such as medicine, pharmacy, nursing and midwifery would be the best idea because everyone would be together and able to express their thoughts.* (midwife, MIDSON 24Oct2021)

*The patients, on the other hand, will not be pleased until they obtain antibiotics. They are unaware of the concepts of sensitivity and resistance, and believe they are not receiving appropriate treatment. Some of them even go to pharmacies to get antibiotics. As a result, I believe that the training should be targeted toward community members and health professionals, including pharmacists.* (hospital-based nurse, non-profit hospital 14Mar2022)

In terms of the community, a few respondents suggested educating secondary school students. Others advocated for working through mothers’ groups and female community health volunteers (FCHV).

*I think we can focus on school students from grade 8 to 10. If we do so then we can educate and provide awareness to most of the people from the new generation which might change the practice of antibiotic use to some extent…. Nowadays there is a provision of school health nurses especially in government schools. We can provide training to them and they can disseminate the information in the school.* (hospital-based nurse, non-profit hospital 14Mar2022)

*We can also focus on FCHV and mothers’ groups in the community. Nowadays, there are youth clubs as well. We can orient them and they can support us in educating the community.* (midwife, MIDSON 14Mar2022)

*In my opinion, providing training to Female Community Health volunteers at the community level will be more effective. After receiving the training, they can counsel the people in their ward.* (community nurse, PHCC 26Oct2021)

## 4 Discussion

Antimicrobial resistance continues to be a significant global health threat particularly in relation to multidrug resistance (MDR) and extensively drug resistant (XDR) pathogens. During the COVID-19 pandemic, hospital-acquired infections and patient length of stays increased in both LMIC and high-income countries. In addition, misuse of antibiotics during the pandemic for febrile and respiratory illnesses have resulted in higher rates of antibiotic resistance globally (27, 28). While the One Health approach is required to address the interrelated causes of resistance in human, animal, and environmental health, the inappropriate use of antibiotics in health systems and in communities continues to significantly impact AMR.

Within health systems, AMR, AMS, and IPC programs need to be designed from a multidisciplinary approach (13, 22, 29). Nurses and midwives within community and hospital settings provide day-to-day care to patients including delivery of medications, observation for potential adverse events and patient health status, and education on homecare. In addition, throughout low resource settings, nurses’ and midwives’ responsibilities can be inclusive of empiric diagnosis and dispensing of antibiotics and other pharmaceuticals (7). One pillar of the drafted National Action Plan for Antimicrobial Resistance in Nepal is to develop “certified Infection Prevention and Control nurses,” [p. 34] which underscores the relationship between infection prevention and antimicrobial stewardship in the long-term and the need for sustainable training in both fields (30).

While nurses and midwives are engaged in activities which are a part of AMS and IPC programs, these roles are often not well-defined within institutional AMS policies and/or are overlooked in terms of education about AMR and stewardship (31, 32). In the current project, a one-day training program was designed specifically to reinforce the roles of nurses and midwives in AMS and IPC. For the most part, while aware of AMR, the study participants had received little or no training in stewardship except for specific and separate education in IPC. Linking AMS and IPC is an important means to emphasize integrated and interdisciplinary trainings within health systems as part of efforts to combat AMR.

The AMS-IPC program was well received by the participants who came from a range of settings (hospitals, clinics, teaching facilities, colleges). Overall, there was enthusiasm for expanding the program elsewhere in Nepal through multiple systems including the government, nursing and medical schools, and through community-based health education programs. These approaches are consistent with targets in the drafted National Action Plan for Antimicrobial Resistance in Nepal. Participants gained knowledge about AMR, AMS, and IPC and data indicate some changes in perceptions regarding empiric dispensing of antibiotics for common symptoms, e.g., cough, fever, diarrhea.

However, the outcome data also indicate more sustained changes in IPC knowledge compared to AMR/AMS knowledge across participants working in various settings. This is likely because participants reported previous exposure to trainings in IPC particularly in the context of the COVID-19 pandemic. Alternatively, a majority of participants stated that they did not have any previous training specific to AMR and AMS. Therefore, multiple trainings and exposure to educational programs about AMS/AMR need to be institutionalized at both the pre- and post-service levels for nurses, midwives, and other healthcare workers.

Perhaps more promising in terms of implementation of knowledge and sustainable change is through the qualitative interviews when participants talked about not only what they learned but how they translated that knowledge into practice. In addition, many of those interviewed also talked about post-training communication with peers and family about AMR and misuse of antibiotics.

Through the process evaluation, the study team also heard respondents’ perceptions about institutional and structural barriers to implementation of AMS programs including hierarchical structures within health facilities, challenges with management and staff reluctance to make changes in prescribing protocols, lack of official prescribing guidelines, and limited enforcement of policies related to dispensing of antibiotics without prescription. These high-level challenges can impede health workers motivations to make and implement changes within their workplace. AMS and IPC policies and programs must be at the forefront of national and local government and hospital and clinic administration priorities to ensure decreases in resistance and effectiveness of antibiotics to treat community- and hospital-acquired infections.

Many trainees expressed the need for a more system-wide approach to AMS that would include education of all health workers including physicians, nurses, midwives, paramedics, and pharmacists, as well as education of female community health volunteers (FCHV) and the general community. Literature suggests a significant lack of AMS-IPC education throughout health systems in both LMIC and higher income countries. In a cross-sectional global survey of hospital health workers including 39 LMIC, only 17% reported regular/required education on AMS and 25% on IPC (33).

When resources are limited, there is increased need for comprehensive, evidence-based training to decrease the spread of multidrug-resistant organisms, consistent with best practices for antimicrobial stewardship programs. Additional longitudinal studies are needed to understand the full effect of training programs in AMS and IPC on patient outcomes in settings where trained infection preventionists may not be feasible, such as rural or staff-limited hospitals or primary healthcare clinics in LMIC. Development and adaptation of AMS-IPC education programs are not the only step to address the global threat of AMR; however, education is an essential first step to support advocacy, communication, and change at the local, national, and international levels.

### 4.1 Study facilitators and limitations

The AMS-IPC training program was delayed due to COVID-19. However, throughout that period, Henry Ford Health and local partners were in communication to develop and adapt the training content. In addition, the training program was organized to meet COVID-19 restrictions in Nepal. Despite these challenges, enrolment and participation met the planned goal of 125 attendees. Through the local partnership, the study also attained a 100% retention for the longitudinal quantitative evaluation.

The training only included nurses living in Kathmandu Valley. This is an urban area with many more health resources than rural and remote regions elsewhere in Nepal. Therefore, results from the study may not be generalizable to other areas of Nepal. The study design did not include a control group or randomization which limits the ability to assess the impact of the program. Further research is needed to determine how AMR stewardship and IPC programs can feasibly be implemented in a variety of health facilities in Nepal including government, non-profit, and private hospitals, and primary health care clinics.

## 5 Conclusion

The AMS-IPC training increased knowledge and decreased intentions for dispensing antibiotics. Participants provided concrete examples of implementation of learnings into practice and identified barriers to AMS and IPC programs. Future dissemination of the training will be adapted to address challenges and content will be modified to further meet the needs of nurses and midwives.

## Data Availability

The datasets presented in this study can be found in online repositories. The names of the repository/repositories and accession number(s) can be found at: OpenICPSR (https://doi.org/10.3886/E204582V1).
